# Chitosan’s Ability to Remove the Smear Layer—A Systematic Review of Ex Vivo Studies

**DOI:** 10.3390/medicina61010114

**Published:** 2025-01-14

**Authors:** Ana Ferreira-Reguera, Inês Ferreira, Irene Pina-Vaz, Benjamín Martín-Biedma, José Martín-Cruces

**Affiliations:** 1 Faculty of Dentistry, University of Santiago de Compostela, 15782 Galicia, Spain; anaferreirareguera@gmail.com (A.F.-R.); pepe3214@gmail.com (J.M.-C.); 2 Faculty of Dental Medicine, University of Porto, 4200-393 Porto, Portugal; inesrvferreirta5@gmail.com (I.F.); igvaz@fmd.up.pt (I.P.-V.); 3 CINTESIS, Faculty of Medicine, University of Porto, 4200-319 Porto, Portugal

**Keywords:** chitosan, smear layer, chelator, irrigant, systematic review

## Abstract

*Background and Objectives*: This systematic review aimed to compare the effect of chitosan in smear layer removal with other commonly used chelators during root canal treatment. *Materials and Methods*: The PRISMA guidelines were followed. Ex vivo studies performed in non-endodontically treated extracted human permanent teeth with a fully formed apex, in which sodium hypochlorite was the main irrigant and chitosan was used as final irrigation to observe its capacity to remove the smear layer using a Scanning Electron Microscope (SEM), were included. In addition, reviews, letters, opinion articles, conference abstracts, book chapters, or articles that did not use a control group were excluded. A literature search was undertaken without limits on time or language, until February 2024, in PubMed—MEDLINE, Scopus, Web of Science, and in the electronic archives of four endodontic journals. The risk of bias was evaluated by adapting the risk of bias assessment used in a previous study. Study selection, data collection, and synthesis were performed and the risk of bias was assessed by two independent reviewers. *Results*: Six studies fulfilled the eligibility criteria and were included. Four studies found chitosan to be as effective as EDTA and one paper showed it was more effective than EDTA and MTAD; however, one article found it to be comparable to citric acid. The overall risk of bias was medium. Quantitative analysis of the results was not possible due to the heterogeneity found between the study methodologies of the included articles. *Conclusions*: Within the limitations of this study, 0.2% chitosan may be considered as a promising irrigation solution when employed as a final irrigant in order to remove the smear layer. Nonetheless, a standardized protocol for the use of chelators in root canal treatment should be established in future studies.

## 1. Introduction

During endodontic treatment, the use of cutting instruments leads to the formation of the smear layer [1], which is formed by organic and inorganic components [2], mainly pulp tissue debris [3], bacteria and their by-products, and mineralized tissue debris [4].

There has always been controversy in the literature over whether to keep or to remove the smear layer. Some authors claimed that maintaining this layer may prevent bacteria and their by-products from entering the dentinal tubules [5]. However, nowadays more studies support the removal of the smear layer [2,5,6,7]. Greater disinfection of the root canal is achieved when the entrance of irrigants and medications into the dentinal tubules is allowed [8]. Nikhil et al. affirms that the smear layer does not allow an adequate seal [9]. Consequently, if the smear layer is removed, more contact between sealers, fillings, and the root canal walls is achieved [1], enabling the penetration of sealers into the dentinal tubules and avoiding microleakage [3].

Several irrigants have been used to remove the smear layer, but none have been able to remove it completely [10]. The literature suggests the use of sodium hypochlorite (NaCl) in order to dissolve the organic portion of the smear layer [2]. On the other hand chelators such as ethylenediaminetetraacetic acid (EDTA), citric acid (CA), or MTAD can be used to demineralize the inorganic content [11].

Although EDTA is considered the gold-standard chelator, it can cause dentin erosion, inactivate sodium hypochlorite [2], and reduce the microhardness of dentin [1,12], and it is considered a pollutant [9,13]. Nonetheless, CA has shown low toxicity [5] and better results than EDTA at smear layer removal according to Shekhar et al. However, other studies have not found statistical differences between the action of CA and EDTA on the smear layer [14] and a similar outcome has been seen for reducing dentin microhardness [15]. Another irrigant is MTAD (mixture of tetracycline, an acid, and a detergent) which has a role in eliminating the smear layer [7] and presents low cytotoxicity [16]. Still, other irrigants seem to show better smear layer removal effects than MTAD [17].

Nowadays, less aggressive and more natural chelating agents are in demand [2,13]. Chitosan (CH) is a copolymer obtained through alkaline’s partial deacetylation of chitin, which is found in crustacean and shrimp shells [1,13]. This polysaccharide is biocompatible, biodegradable, bioadhesive, and non-toxic [1,2,8,9,13]. Its use has been proposed in different dentistry fields, as it shows wound-healing abilities, hemostatic capacity, periodontal anti-inflammatory activity, and bone repair properties in in vitro studies [18]. Regarding endodontics, chitosan has been suggested as an irrigant as it has antimicrobial properties [2,8] and chelating abilities and is low cost [1,9]. Da Cruz-Filho et al. found that a 0.2% chitosan solution can maintain its chelating properties for a period of at least 6 months [13]. Its mechanism of action is not completely clear, but adsorption, ionic exchange, and chelation are thought to be responsible for the creation of complexes between the chemical agent and the metallic ions [1,12,13]. Some studies show that a final irrigation with CH has the ability to remove the smear layer due to its metallic ion binding ability [2], even at low concentrations [8]. CH has been proposed as an alternative to other chelators like EDTA or citric acid, for its similar smear layer removal ability which causes less dentin erosion [8,9,13].

Ilhan et al. recently published a systematic review and meta-analysis of in vitro studies regarding the smear layer removal ability of chitosan compared to EDTA, reporting similar results for both compounds [19]. Another systematic review in which in vitro studies comparing chitosan with other irrigants were included, concluded that chitosan has better results than citric acid, similar results to MTAD, but when compared with EDTA the results were not conclusive [20]. The aim of this systematic review was to evaluate chitosan’s ability on smear layer removal, specifically in comparison to other commonly employed root canal chelating irrigants, in order to offer insights for future clinical application.

## 2. Materials and Methods

This systematic review was registered in the PROSPERO database (PROSPERO; Centre for Reviews and Dissemination, University of York), under the number CRD42024532945, and was reported following the recommendations of the Preferred Reporting Items for Systematic Review and Meta-Analysis (PRISMA 2020) guidelines [21].

### 2.1. Eligibility Criteria

This study was conducted to answer the following PICO (problem, intervention, comparison, outcome) question: In teeth undergoing root canal treatment (P), does the use of chitosan as a root canal irrigant (I) effectively remove the smear layer (O) compared to other commonly used root canal chelating irrigants (C)?

Ex vivo studies performed in non-endodontically treated extracted human permanent teeth with a fully formed apex, in which sodium hypochlorite was the main irrigant and chitosan was used as final irrigation to observe its smear layer removal ability using a Scanning Electron Microscope (SEM), were included. In addition, reviews, letters, opinion articles, conference abstracts, book chapters, or articles that did not use a control group were excluded. The exclusion and inclusion criteria are specified in Table 1.

### 2.2. Search Strategy

A literature search was performed in February 2024 on the PubMed, Scopus, and Web of Science electronic databases. Also, we carried out an additional screening on the references of the selected studies, a search of the gray literature through OpenGrey, and a manual search realized in the electronic archives of the *Journal of Endodontics, International Endodontic Journal, European Endodontic Journal,* and *Australian Endodontic Journal* for additional papers. Different keywords were combined with the Boolean operators “AND” and “OR” to carry out the electronic database search. The electronic research strategy is shown in Table 2.

The references obtained from the different databases were imported into Zotero Software 6.0.36 (Corporation for Digital Scholarship and the Roy Rosenzweig Center for History and New Media, George Mason University, USA) and duplicates were removed.

### 2.3. Selection Process and Data Collection

Titles and abstracts were scanned by two independent researchers, and a selection was made following the inclusion and exclusion criteria specified above by each investigator. If the available information in the title or abstract was not enough to confirm the inclusion or exclusion of an article, the full article was read.

The parameters chosen to extract information from the included articles were as follows: type of teeth, sample size per group, apical diameter, main irrigant used during instrumentation, groups according to the final irrigation, volume of final irrigation and time of usage, type of analysis realized, and main results obtained. A table was made to collect the most important information from each article. If any information was not clearly mentioned in the studies, NM (not mentioned) was written. Two authors independently extracted the data from the included studies. Disagreements were resolved by a third author during the selection process and data collection.

### 2.4. Risk of Bias Assessment

The risk of bias was assessed according to an adaptation of the Cochrane criteria for ex vivo studies and similar to previous reviews [22,23]. The analyzed parameters were (1) standardization of sample selection (type of teeth), (2) sample size calculation, (3) randomization, (4) blinding, (5) standardized preparation (single operator), and (6) reporting of data. The risk of bias was considered low (−) if the selected parameters were mentioned, but high (+) if the authors did not mention the parameters. Each included study was independently evaluated by two authors. Disagreements were resolved by a third author.

A study was considered at a low risk of bias if five or six of the six criteria assessed were reported. If three or four criteria were reported, the study was judged as having a medium risk of bias. Those reporting one or two criteria were considered at a high risk of bias.

## 3. Results

### 3.1. Selection of the Studies

**Figure 1:**
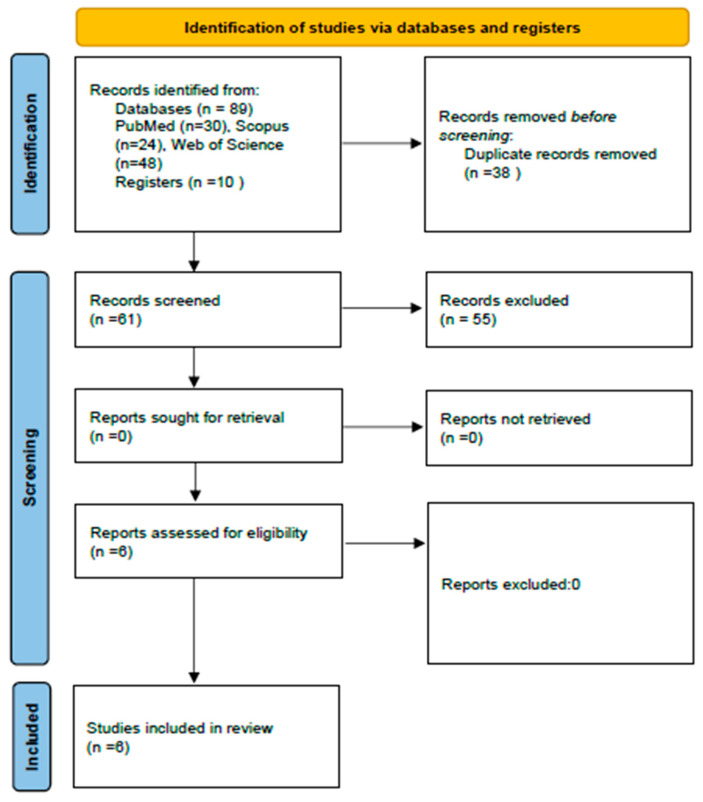
PRISMA flow diagram search.

The results from the literature search process are shown in Figure 1. The search strategy generated 102 studies. After duplicates were removed, 61 papers were selected for title and abstract screening. In total, 55 studies were excluded. Finally, 6 studies fulfilled the eligibility criteria and were included in this systematic review [24,25,26,27,28,29].

### 3.2. Data Collection

The principal findings of the included studies are summarized in Table 3.

#### 3.2.1. Type of Teeth and Sample Size

In the included studies, the type of teeth used were different from one another: Straight single-rooted Vertucci’s type I [24], single-rooted [25], single-rooted mandibular premolars [26], incisors [27], maxillary canines [28], and single-rooted premolars [29]. A sample size of 10 teeth was determined in three articles [25,27,29]. The other sample sizes varied from 5 [28] to 12 teeth [24].

#### 3.2.2. Irrigation Protocols

Sodium hypochlorite (NaOCl) was the main irrigant used in all of the included papers, following the inclusion criteria. However, its concentration varied from 1% [28] to 5.25% [27]. The final irrigants used included chitosan at 0.2%, following the inclusion criteria [24,25,26,27,28,29]. Additional experimental groups used 17% EDTA [24,25,26,27,28], 10% citric acid [28], 2.5% NaOCl [26], 1% acetic acid [28], and MTAD [29].

#### 3.2.3. Type of Chitosan Used

The type of chitosan used was not mentioned in two of the included articles [25,29]. In two other papers, chitosan with a 90% degree of deacetylation [28] or higher than 90% [27] was used. Additionally, chitosan powder with a 75% [24] or 90% degree of deacetylation [26] was selected.

#### 3.2.4. Volume and Time of Irrigation

A total of 5 mL was the volume selected for the final irrigation in all of the included studies, during a time of irrigation of 1 [27], 3 [24,26,27,28,29], or 5 [25] min.

#### 3.2.5. Size of Apical Preparation

The size of the final apical preparation was not mentioned in three [27,28,29] of the six included studies. In the other three articles, the apical size varied from ISO #30 [24,26] to ISO #35 [25].

#### 3.2.6. Selection of Irrigation Needle and Activation

The needles selected for the irrigation process were diverse: 31 G [24], sterile 30 G [26], and 0.45 × 13.0 mm [28]. On the other hand, three articles did not mention the type of needle that was used [25,27,29]. Activation was only mentioned to be ultrasonic by Kamble et al. [25].

#### 3.2.7. Method of Smear Layer Assessment

Although all of the included articles used SEM, this being an inclusion criterion, the magnification varied from 350× [28] to 3000× [25], through 1000× [25,26,27,29] and 2000× [24,26,29]. Half of the teeth samples were observed by Abdelkafy et al. [24], Silva et al. [28], and Zhou et al. [29], while the other three authors [25,26,27] observed both under SEM. Abdelkafy et al. took images from the middle of each third but did not specify the methodology for assuring the exact position where the photograph should be taken [24]. On the other hand, Silva et al. marked the samples to standardized the locations where the representative images were to be obtained [28]. In the other four studies, photographs of the “representative areas” of the thirds studied were captured [25,26,27,29]. In three articles, all thirds of the root canals were observed [24,27,29], while other two only selected the apical part [24,26], and Silva et al. obtained results from half of the middle and apical thirds [28].

#### 3.2.8. Comparison of the Chitosan with Other Irrigants

In four studies, the effectiveness of chitosan was comparable to EDTA [24,26,27,28], while one study showed chitosan was more effective than EDTA [25] and MTAD [29]. In one study, chitosan showed a comparable effect to citric acid [28].

### 3.3. Risk of Bias

Table 4 shows the risk of bias results of the included studies. None of the selected studies had a low risk of bias in all parameters evaluated [24,25,26,27,28,29]. There was a low risk of bias regarding the standardization of sample selection in all of the included studies, as they reported this parameter [24,25,26,27,28,29]. Sample size calculation was only mentioned by Abdelkafy et al. [24] and Sehitoglu et al. [27]. Four studies reported that the samples were randomly assigned to the experimental and control groups, being classified as “low risk” in this domain [24,25,27,28]. All studies had a high risk of bias on blinding (the evaluator(s) was not blinded to the experimental protocols) and for standardization of the preparation (studies did not report whether the experimental procedures were performed by a single operator) [24,25,26,27,28,29]. All studies reported the data obtained and were therefore considered to have a low risk of bias [24,25,26,27,28,29].

## 4. Discussion

The ideal irrigation protocol must address the biological and mechanical or deleterious effect on dentin [30]. The recommended irrigation protocol for the successful removal of both organic and inorganic components of the smear layer is sodium hypochlorite, followed by chelators, mainly EDTA or citric acid [30,31]. The effect of a chelating agent depends mainly on its concentration, amount of solution, and application time [32]. Except for Kamble et al., who settled on a time of application of 5 min [25], all of the included articles had one experimental group that used 5 mL of 0.2% chitosan as a final irrigant for 3 min [24,26,27,28,29].

Although acceptable results can be achieved with EDTA, it has been shown that it can influence inflammatory reactions and periapical healing [33], cause dentin erosion [34], and increase the likelihood of perforation during instrumentation [35]. Similarly, citric acid has demonstrated effectiveness in the removal of the smear layer [1]. However, its use leads to peritubular and intertubular dentin erosion, with a consequent reduction in its microhardness [36]. Although MTAD exhibits action in smear layer demineralization, and it does not seems to damage the dentin structure [37], EDTA obtains better results than MTAD in smear layer removal [38].

Chitosan, a natural polysaccharide, has attracted attention in endodontics due to its biodegradability, bioadhesive, biocompatibility, antibacterial properties, and lack of toxicity, showing promising results in smear layer removal [10,39]. Two hypotheses can be found in the literature as an attempt to explain the chelating mechanism of chitosan: the “bridge model” is based on the linking of two or more amino groups of chitosan to the same metal ion, while the “pendant model” refers to the binding of only one amino group with a metal ion [31,40,41,42]. It is not clear which one of these processes is behind the calcium ion chelation that results into the degradation of inorganic matter from the smear layer [40,42]. In addition, chitosan phosphate groups might bind to calcium ions, this action induces the remineralization of demineralized root canal dentine [40,41]. There is some controversy regarding the effect of chitosan irrigation on dentin microhardness: while some authors claim it alters dentin properties by lowering its microhardness [43], others show that chitosan causes less dentin erosion when compared to EDTA [34]. Veeraiyan et al. recently demonstrated that the smear layer removal capacity of 0.2% chitosan was equal to that of EDTA, with limited roughness [31]. Bastawy et al. showed that 0.2% chitosan presented lower alteration of dentin microhardness when compared to 17% EDTA [44]. Regarding the smear layer removal ability of chitosan, according to the studies included in this review, 0.2% chitosan as a final irrigant has demonstrated similar or better results when contrasted to other commonly used chelators [24,25,26,27,28,29].

Specifically, it can be found that 0.2% chitosan exhorted a better chelating action than MTAD at the apical region (with no statistical differences between the three thirds of the canal) [29] and comparable smear layer removal results to 15% EDTA, 17% EDTA, or 10% citric acid on the different regions of the root canals [24,26,27,28]. However, Abdelkafy et al. found similar outcomes when using 17% EDTA or 0.2% chitosan on the coronal third, while differences were cleared between both chelators in regard to their action on the middle and apical thirds, with 17% EDTA statistically being more effective [24]. Sehitoglu et al. suggest this can be due to the SEM observation of the samples, since the representative image is randomly obtained from each area, and it can be deceiving when interpreting the results obtained [27]. In addition, it is not possible to compare the results obtained based on the type of chitosan used, due to the lack of uniformity regarding the degree of deacetylation of the chitosan employed on the included studies.

One limitation of this study was the high risk of bias on several items of the assessment tool, like blinding and the standardization of specimen preparation. These parameters must be explicitly reported in order to keep the risk of bias low. In any case, a quantitative analysis was not feasible due to the heterogeneity of the study methodologies. The variability between methodologies showed that authors should focus on the better description and design of experimental studies. Furthermore, the results of this review should be interpreted with caution due to the lack of studies on this topic.

The preparation of the samples for SEM observation (mounting, sectioning, and gold sputtering) may lead to modifications on the debris remaining on the surfaces [19]. In addition, the image selected might not be clearly representative of the studied third, since some dentine areas are not reached during instrumentation (hence, no smear layer is produced) and closed tubules in sclerotic dentine can mislead the results due to the appearance of the smear layer [19]. It can be deduced that the higher the zoom applied in the images, the clearer the observed zones will be; that is why the results from a visual score in a x350 image cannot be compared to the ones from a ×3000 image. Therefore, scoring results from visual observation of differently augmented images are difficult to compare. From the observation of the samples, Kamble et al. concluded that the chitosan solution was the irrigant that better removed the smear layer from the apical third at x1000 and ×3000 magnification [25], while others, like Abdelkafy et al. or Sehitoglu et al., affirmed that the chitosan solution was as effective as EDTA at removing the smear layer, at ×2000 and ×1000 magnification, respectively [24,27]. From the included studies, only Silva et al. specified the protocol for obtaining the images at the center of each third via SEM [28]. However, it cannot be assured that those specific areas were touched by the files through the mechanical preparation. Comparison of the images of the root canal surface before and after the root canal treatment protocol selected may be of assistance to verify the process and the results [45].

The following were perceived as the strongest points of the present review: (1) an a priori review protocol was prepared and registered in the PROSPERO database; (2) a comprehensive literature research was conducted in four electronic databases, with no language restriction, and including the gray literature; and (3) the authors followed the recommendations of the Preferred Reporting Items for Systematic Review and Meta-Analysis (PRISMA 2020) guidelines [21].

However, given the circumstances, in all of the studies using chitosan as a final irrigant, the results show similar, if not better, results in smear layer removal for chitosan, when compared to the other quelants. That being said, the findings of the present systematic review should be interpreted cautiously. Future studies should be directed toward establishing a protocol for the standardized use of chelators in endodontics. Randomized controlled studies are necessary to assess the effect of chitosan on smear layer removal compared to the decalcifying agents commonly used.

## 5. Conclusions

Within the limitations of this study, 0.2% chitosan may be considered a promising solution to use as a final rinse to remove the smear layer.

## Figures and Tables

**Table 1:** Inclusion and exclusion criteria.

Inclusion Criteria	Exclusion Criteria
Ex vivo studies	Reviews
Non-endodontically treated extracted human teeth	Letters
Permanent teeth with fully formed apex	Opinion articles
Sodium hypochlorite as main irrigant	Conference abstracts
Chitosan as final irrigant	Book chapters
SEM to observe smear layer removal	Articles with no control group

**Table 2:** Database search strategy.

Database	Search Strategy	Findings
PubMed	#1 ((chitosan[Title/Abstract]) OR (chitosan[MeSH Terms])	
	#2 ((smear layer[Title/Abstract])) OR (smear layer removal[Title/Abstract]) OR (chelator[Title/Abstract]) OR (chelators[Title/Abstract]) OR (final irrigation[Title/Abstract]) OR (final irrigant[Title/Abstract]) OR (final irrigants[Title/Abstract]) OR (smear layer[MeSH Terms]) OR (chelator[MeSH Terms])	
	#3 ((endodontic[Title/Abstract]) OR (endodontics[Title/Abstract]) OR (root canal therapy[Title/Abstract]) OR (root canal treatment[Title/Abstract]) OR (endodontic therapy[Title/Abstract]) OR (endodontic treatment[Title/Abstract]) OR (root canal treatment[MeSH Terms]) OR (endodontics[MeSH Terms])	
	#1 and #2 and #3	30
Scopus	#1 TITLE-ABS-KEY(“chitosan”)	
	#2 TITLE-ABS-KEY(“smear layer” or “smear layer removal” or “chelator” or “chelators” or “final irrigation” or “final irrigant” or “final irrigants”)	
	#3 TITLE-ABS-KEY(“endodontic” or “endodontics” or “root canal therapy” or “root canal treatment” or “endodontic therapy” or “endodontic treatment”)	
	#1 and #2 and #3	24
Web of Science	#1 TS=((chitosan))	
	#2 TS=((smear layer) OR (smear layer removal) OR (chelator) OR (chelators) OR (final irrigation) OR (final irrigant) OR (final irrigants))	
	#3 TS=((endodontic) OR (endodontics) OR (root canal therapy) OR (root canal treatment) OR (endodontic therapy) OR (endodontic treatment))	
	#1 and #2 and #3	48

**Table 3:** Extracted information from the included articles.

**Main Results**	“0.2% CH and CNP have comparable chelating effects to 17% EDTA and induce remineralization of the root canal dentin”	“0.2% CH removes SL ^14^ with greater efficiency than 17% EDTA in a third of root canals”	“0.2% CH had same effect on SL removal compared to 17% EDTA”	“There was no significant difference between EDTA and CH solutions in all three regions of the tooth”	“The 0.2% CH solution was able to remove SL and provide statistically similar results to 15% EDTA and 10% CA”	“CH was more effective in SL removal than MTAD”
**Analysis Method**	SEM ^10^ ×2000 1 half. C ^11^, M ^12^, A ^13^. 2 blinded observers	SEM ×1000 ×3000. Both halves. A. Blinded examiner	SEM ×1000 ×2000 both halves. A. 2 blinded examiners	SEM ×1000 Both halves. C, M, A. 2 blinded researchers	SEM ×350 1 half. Half M and A. 3 endodontic observers	SEM ×1000, ×2000. 1 half. C, M, A. 3 investigators
**Activation**	NM ^9^	Ultrasonic	NM	NM	NM	NM
**Time FI**	3′	5′	3′	G1: NM G2, G4, G5, G6: 3′ G3: 1′	3’	3’
**Volume FI and Needle**	5 mL 31-gauge needle	5 mL Needle NM	5 mL sterile 30 G needle	5 mL Needle NM	5 mL 0.45 × 13.0 mm needle	5 mL Needle NM
**Type of Chitosan**	Chitosan powder (90% deacetylation)	NM	Chitosan powder (>75% deacetylation)	Chitosan (>90% deacetylation)	Chitosan (90% deacetylation)	NM
**Groups FI ^1^**	2 CG: (GIA: saline, GIB: NP ^4^ + NT ^5^). GII: 0.2% CH ^6^; GIII: 0.2% CNP ^7^, GIV: 17% EDTA ^8^	G1: 17% EDTA, G2: CG NT; G3: 0.2% CH; G4: NT	G1: 17% EDTA, G2: 0.2% CH, G3: CG 2.5% NaOCl	G1 (CG): DW ^15^; G2: GO ^16^; G3: 17% EDTA; G4: 0.2% CH; G5: GO-EDTA; G6: GO-CH	G1: 15% EDTA; G2: 0.2% CH; G3: 10% CA ^17^; G4: 1% AA ^18^; G5: CG (no FI).	G1: (CG) saline; G2: 0.2% CH; G3: MTAD ^19^
**Main Irrigant**	2.6% NaOCl ^3^	3% NaOCl	2.5% NaOCl	5.25% NaOCl	1% NaOCl	5.25% NaOCl
**Apical Diameter**	X3—30	35	30 (F3)	NM	NM	NM
**Sample Size per Group**	12 CG ^2^ n = 6	10	8	10	5	10
**Type of Teeth**	Straight single-rooted Vertucci’s type I	Single-rooted	Single-rooted mandibular premolars	Incisor	Maxillary canines	Single-rooted premolars
**Author and Year**	Abdelkafy, 2023 [24]	Kamble, 2017 [25]	Ratih, 2020 [26]	Sehitoglu, 2023 [27]	Silva, 2013 [28]	Zhou, 2018 [29]

^1^ Final irrigant. ^2^ Sodium hypochlorite. ^3^ Control group. ^4^ No preparation. ^5^ No treatment. ^6^ Chitosan. ^7^ Chitosan nanoparticles. ^8^ Ethylenediaminetetraacetic acid. ^9^ Not mentioned. ^10^ Scanning Electron Microscope. ^11^ Coronal third. ^12^ Medium third. ^13^ Apical third. ^14^ Smear layer. ^15^ Distilled water. ^16^ graphene oxide. ^17^ Citric acid. ^18^ Acetic acid. ^19^ Mixture of tetracycline, an acid and a detergent.

**Table 4:** Risk of bias.

	Standardization of Samples Selection (Type of Teeth)	Sample Size Calculation	Randomization	Blinding	Standardized Preparation (One Single Operator)	Reporting of Data	Result of Risk of Bias Evaluation
Abdelkafy, 2023 [24]	+	+	+	−	−	+	medium
Kamble, 2017 [25]	+	−	+	−	−	+	medium
Ratih, 2020 [26]	+	−	−	−	−	+	high
Sehitoglu, 2023 [27]	+	+	+	−	−	+	medium
Silva, 2012 [28]	+	−	+	−	−	+	medium
Zhou, 2018 [29]	+	−	−	−	−	+	high

## Data Availability

No additional data are available since this is a protocol for a systematic review.
