# Correction: Ferreira-Reguera et al. Chitosan’s Ability to Remove the Smear Layer—A Systematic Review of Ex Vivo Studies. *Medicina* 2025, *61*, 114

**DOI:** 10.3390/medicina62040749

**Published:** 2026-04-14

**Authors:** Ana Ferreira-Reguera, Inês Ferreira, Irene Pina-Vaz, Benjamín Martín-Biedma, José Martín-Cruces

**Affiliations:** 1 Faculty of Dentistry, University of Santiago de Compostela, 15782 Galicia, Spain; anaferreirareguera@gmail.com (A.F.-R.); pepe3214@gmail.com (J.M.-C.); 2 Faculty of Dental Medicine, University of Porto, 4200-393 Porto, Portugal; inesrvferreirta5@gmail.com (I.F.); igvaz@fmd.up.pt (I.P.-V.); 3 CINTESIS, Faculty of Medicine, University of Porto, 4200-319 Porto, Portugal; 4 Oral Sciences Research Group, Endodontic and Restorative Dentistry Unit, School of Medicine and Dentistry, Universidade de Santiago de Compostela, Health Research Institute of Santiago (IDIS), 15705 Santiago de Compostela, Spain

## Additional Affiliation

In the published publication [1], there was an error regarding the affiliation for Benjamín Martín-Biedma. In addition to Affiliation 4, the updated affiliation should include the following: Oral Sciences Research Group, Endodontic and Restorative Dentistry Unit, School of Medicine and Dentistry, Universidade de Santiago de Compostela, Health Research Institute of Santiago (IDIS), 15705 Santiago de Compostela, Spain. The authors state that the scientific conclusions are unaffected. This correction was approved by the Academic Editor. The original publication has also been updated.
